# Biallelic *CDK9* variants as a cause of a new multiple-malformation syndrome with retinal dystrophy mimicking the CHARGE syndrome

**DOI:** 10.1038/s10038-021-00909-x

**Published:** 2021-02-27

**Authors:** Sachiko Nishina, Katsuhiro Hosono, Shizuka Ishitani, Kenjiro Kosaki, Tadashi Yokoi, Tomoyo Yoshida, Kaoru Tomita, Maki Fukami, Hirotomo Saitsu, Tsutomu Ogata, Tohru Ishitani, Yoshihiro Hotta, Noriyuki Azuma

**Affiliations:** 1 Department of Ophthalmology and Laboratory for Visual Science, National Center for Child Health and Development, Tokyo, Japan; 2 Department of Ophthalmology, Hamamatsu University School of Medicine, Shizuoka, Japan; 3 Department of Homeostatic Regulation, Research Institute for Microbial Diseases, Osaka University, Osaka, Japan; 4 Center for Medical Genetics, Keio University School of Medicine, Tokyo, Japan; 5 Heiwa Eye Clinic, Tokyo, Japan; 6 Department of Molecular Endocrinology, National Research Institute for Child Health and Development, Tokyo, Japan; 7 Department of Biochemistry, Hamamatsu University School of Medicine, Shizuoka, Japan; 8 Department of Pediatrics, Hamamatsu University School of Medicine, Shizuoka, Japan

**Keywords:** Medical genetics, Medical research

## Abstract

*CDK9* has been considered a candidate gene involved in the CHARGE-like syndrome in a pair of cousins. We report an 8-year-old boy with a strikingly similar phenotype including facial asymmetry, microtia with preauricular tags and bilateral hearing loss, cleft lip and palate, cardiac dysrhythmia, and undescended testes. Joint contracture, no finger flexion creases, and large halluces were the same as those of a previously reported patient with homozygous *CDK9* variants. The ocular phenotype included blepharophimosis, lacrimal duct obstruction, eyelid dermoids, Duane syndrome-like abduction deficit, and congenital cataracts. Optical coherence tomography and electroretinography evaluations revealed severe retinal dystrophy had developed at an early age. Trio-based whole-exome sequencing identified compound heterozygous variants in *CDK9* [p.(A288T) of maternal origin and p.(R303C) of paternal origin] in the patient. Variants’ kinase activities were reduced compared with wild type. We concluded that *CDK9* biallelic variants cause a CHARGE-like malformation syndrome with retinal dystrophy as a distinguishing feature.

## Introduction

Cyclin-dependent kinase 9 (CDK9) regulates cellular transcription, growth, and proliferation following binding to cyclin T1 and forming the positive transcription elongation factor b complex. CDK9 expression is ubiquitous, including involvement in the development and growth of cardiac cells, hepatocytes, hematopoietic tissue, adipocytes, neurons, and muscle cells. Recent studies have highlighted its direct and indirect involvement in pathological pathways of diverse biologic processes including cancers, cardiovascular diseases, viral replication, and morphogenesis [1]. In zebrafish, CDK9 regulates larval morphogenesis including the brain, heart, eye, and blood vessels [2], and in mice, complete loss-of-function mutations in *Cdk9* were lethal and heterozygote loss of function was associated with abnormal morphology of the heart, skin, and epididymis [3].

The roles of *CDK9* in human morphogenesis have not been established. A pair of cousins from a single consanguineous family has been reported to have CHARGE syndrome-like features including coloboma, renal malformation, restricted growth, and limb anomalies with a rare variant of *CDK9* [4]. The cousins, who were ascertained through exome analysis and autozygosity mapping of patients with various multiple anomalies and were born to highly consanguineous parents, had a homozygous nonsynonymous variant p.Arg225Cys in *CDK9*. It remains unclear whether the homozygosity of that variant contributed to the CHARGE syndrome-like phenotype or the association occurred by chance. Indeed, the authors of the article labeled *CDK9* as the “novel candidate gene” but not as the causative gene. Recently, three additional patients from three families with the same homozygous variant, p.Arg225Cys, in *CDK9* also were reported to have CHARGE syndrome-like features [5]. Demonstration of biallelic pathogenic variants of *CDK9* in “multiple unrelated individuals or families affected by the same very rare but highly recognizable clinical condition” confirmed the causality [6]. We report a patient with new compound heterozygous pathogenic rare variants who fulfilled this criterion of FORGE Strategies for Gene Discovery, establishing *CDK9* biallelic deficiency as a novel cause of human multiple-malformation syndrome involving the eyes with a vision-threatening retinal dystrophy.

## Patient report

The proband was an 8-year-old boy who was referred to the Department of Ophthalmology, National Center for Child Health and Development (NCCHD) with suspected visual impairment and nyctalopia of 2-year duration. He was born at 39 weeks gestational age to healthy nonconsanguineous parents and weighed 2989 g.

**Fig. 1:**
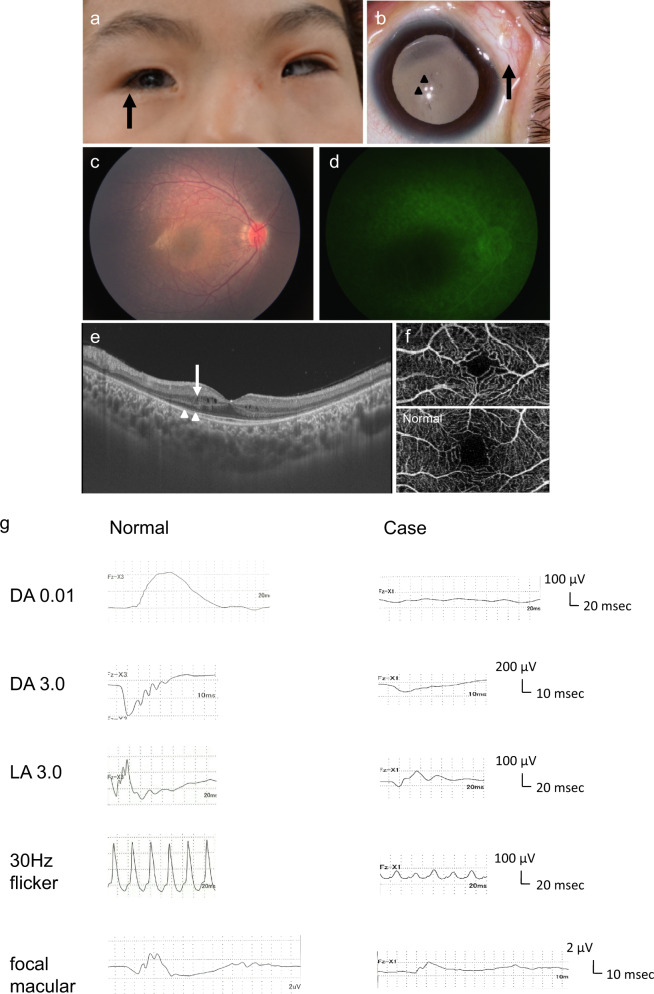
The photograph demonstrates the ophthalmic phenotype of the patient. **a** Blepharoconjunctivitis due to lacrimal duct obstruction is shown. The right eye cannot abduct past the midline (arrow). **b** Bilateral cataracts are present (arrowheads) and dermoids are seen in the lateral canthus of the left eyelid (arrow). **c** A fundus photograph of the right eye shows extensive retinal degeneration including the macula. **d** A fluorescein angiography image shows diffuse retinal pigment epithelium atrophy. **e** A swept-source optical coherence tomography (OCT) image of the right posterior retina shows severe thinning of outer nuclear layer and a diminished ellipsoid zone (arrowheads) except in the foveal area. Cystoid degeneration in the inner nuclear layer is seen in the parafoveal area (arrow). **f** An OCT angiography image of the right foveal zone shows anomalous vascularity in the superficial plexus. The normal image was obtained from a 7-year-old child without retinal disease. **g** Full-field electroretinography (ERG) and focal macular ERG of the right eye of the patient. The rod response (DA 0.01), mixed rod and cone responses (DA 3.0) are diminished, and the cone responses (LA 3.0 and 30 Hz flicker) are reduced markedly. A minimal focal macular response is detected. The normal ERGs were obtained from a 6-year-old child without retinal disease. DA dark adapted, LA light adapted

He had multiple ocular anomalies, i.e., blepharophimosis, bilateral lacrimal duct obstruction, dermoids of the lateral canthus of the left eyelid, Duane syndrome-like abduction deficit in the right eye, and cataracts detected by slit-lamp microscopy (Fig. 1a, b). Ophthalmoscopy showed degenerated retinas bilaterally. The decimal best-corrected visual acuity was 0.2 bilaterally. He maintained orthophoria in primary gaze and demonstrated stereopsis of 200 s of arc on the Lang stereo test.

We conducted comprehensive and detailed ophthalmic examinations under general anesthesia. Fundus photography and fluorescein angiography were performed using the RetCam ophthalmic imaging system (Massie Research Laboratories, Inc., Pleasanton, CA, USA). The central retinal architecture was evaluated using swept-source optical coherence tomography (OCT) and OCT angiography (OCTA) (DRI OCT-1, Topcon, Tokyo, Japan). The retinal function was studied using full-field electroretinography (ffERG) using combined recording and analysis systems (Neuropack 8, Nihon Kohden, Tokyo, Japan) in accordance with the International Society for Clinical Electrophysiology of Vision protocol [7]. The foveal function also was studied using focal macular ERG (fmERG) with a stimulus spot 15° in diameter for 100 ms using the combined recording and analysis systems (ER80, Kowa Company, Tokyo, Japan, and Neuropack 8). Ophthalmoscopy and fluorescein angiography showed significant retinal degeneration including the maculas bilaterally (Fig. 1c, d). OCT imaging also showed significantly thinned photoreceptor outer nuclear layers and diminished ellipsoid zones (EZ) bilaterally. In the foveal area, the EZ were detectable but cystoid degeneration in the inner nuclear layer was prominent (Fig. 1e), and OCTA images visualized anomalous vascularity in the superficial plexus (Fig. 1f). ERGs confirmed the definitive diagnosis of severe rod–cone retinal dystrophy with attenuated foveal function bilaterally (Fig. 1g). In the ffERG, the rod response was diminished and the cone response was decreased markedly in both eyes, indicating progressive rod–cone retinal dystrophy. In the fmERG, minimal responses were detected bilaterally.

**Fig. 2:**
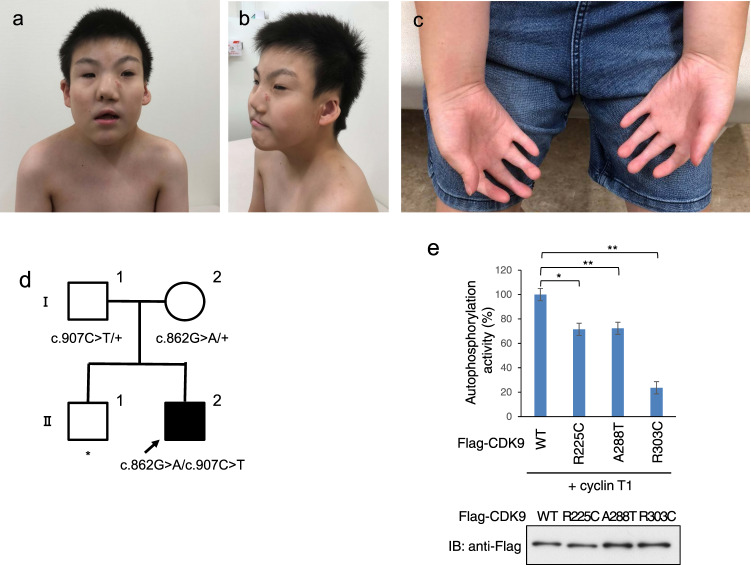
CHARGE-like syndrome phenotypes and genome variation of the patient. The photographs (**a**, **b**) show facial asymmetry, microtia with preauricular tags, cleft lip and palate, narrow forehead, laterally displaced hair whorl, arched eyebrows, epicanthal folds, short palpebral fissures, long eyelashes, low nasal ridge, smooth philtrum, webbed and broad neck, and upsweeping of the posterior nuchal hair line. **c** *CDK9*-related characteristic features of joint contracture, absence of finger flexion creases, and cutaneous syndactyly are seen. **d** The pedigree of a family with *CDK9* variants. The genotypes of proband (II-2) and parents (I-1 and I-2) are shown. The nucleotide numbering reflects cDNA numbering with +1 corresponding to A of the ATG translation initiation codon in the reference sequence NM_001261.3, according to the nomenclature recommended by the Human Genome Variation Society (http://varnomen.hgvs.org/). The initiation codon was designated as codon 1. The proband is indicated by the arrow. Square, male; circle, female; black, disease affected; asterisk, no available DNA sample. **e** In vitro kinase assay of human CDK9. The graph shows the enzymatic activity rates of Flag-tagged CDK9 (Flag-CDK9) wild type (WT), R225C, A288T, and R303C. The error bars indicate the standard deviations of the mean. *p < 0.05, **p < 0.01 using a *t*-test. The amounts of Flag-CDK9 proteins were confirmed by immunoblotting with anti-Flag antibodies (bottom panel)

He presented with multiple malformations: facial asymmetry, microtia with preauricular tags and bilateral hearing loss, cleft lip and palate, cardiac dysrhythmia, undescended testis, and intellectual disability. These features suggested the CHARGE syndrome (Fig. 2a, b). He was developmentally delayed, started to walk unassisted at the age of 3 years, and spoke words with meanings at the age of 7 years. At the age 10 years, he could count to 20 and formed four-word sentences. He had the following minor anomalies: a narrow forehead, laterally displaced hair whorl, arched eyebrows, epicanthal folds, short palpebral fissures, long eyelashes, low nasal ridge, smooth philtrum, webbed and broad neck, upsweeping of the posterior nuchal hair line, hypoplastic nipples, retractile testicle, no finger flexion creases, cutaneous syndactyly, and large halluces (Fig. 2c).

Genomic DNA was extracted from the peripheral blood of the patient and his parents using standard procedures. Trio samples were analyzed by whole-exome sequencing. Exome data processing, variant calling, and variant annotation were performed as described previously [8, 9], using human GRCh38 as the reference genome. To identify disease-causing variants, we focused on nonsense, frameshift variants, nonsynonymous, and splice-site variants within 10 basepairs of the exon–intron boundaries and excluded synonymous and noncoding exonic variants from further analysis. We treated common genetic variants (allele frequency > 0.01 for recessive variants or >0.001 for dominant variants) in any of the ethnic subgroups found in the public databases including the Genome Aggregation Database, Human Genetic Variation Database, and Integrative Japanese Genome Variation Database and in-house exome data (*n* = 218) as putative nonpathogenic sequences. After the filtering steps, we identified two rare missense variants [c.862G>A, p.(A288T) and c.907C>T, p.(R303C)] of the *CDK9* (NM_001261.3) gene. Although the CHARGE syndrome has been reported as an autosomal dominant condition that is caused by the variants in the *CHD7* [10], no de novo rare variants were identified in *CHD7* or any other genes in this study. The *CDK9* variants were validated by Sanger sequencing and cosegregated (Fig. 2d and Supplementary Fig. 1). Both variants are extremely rare, especially the c.907C>T, which was not found in the previously mentioned SNP databases (Table 1). Although only the two variants [c.280C>T, p.(R94C) and c.673C>T, p.(R225C)] have been registered for *CDK9* in the Human Gene Mutation Database to date (Professional version 2020.2, accessed September 23, 2020) [11] and reported to be associated with the intellectual disability with normal visual and hearing [12] and the CHARGE syndrome [4], respectively, the variants detected in this study were novel and have not been reported previously. The amino acid residues A288 and R303 are located in the protein kinases catalytic domain of *CDK9*. The residues were compared with those encoded by orthologous genes in several vertebrates (cow, rat, mouse, chicken, and zebrafish) and found to be highly conserved across different species. In addition, the variants were predicted to be deleterious by in silico pathogenicity prediction tools (Table 1). To examine both variants’ kinase activities, Flag-tagged CDK9 recombinant proteins, wild type (WT), R225C, A288T and R303C, were prepared. Interestingly, CDK9 R225C, A288T, and R303C variants’ kinase activities were reduced compared with WT (Fig. 2e). These results indicate loss of function of CDK9 in the patient. Thus, we evaluated that the two variants are likely to contribute to the phenotype of the patient as disease-causing mutations. While other rare hemizygous and compound heterozygous variants were identified, no data were available to support the relevance of the variants to our patient’s phenotype (Supplementary Table 1).

**Table 1:** The candidate list of the compound heterozygous variants

Identified variant						Frequencies in the databases^a^				Results of the in silico analyses^b^			
Gene (cytoband)	Accession number	Identified variant	Location in gene	Inheritance	Conservation across species^c^	In house (*n* = 218)	HGVD	4.7KJPN	gnomAD	SIFT	Polyphen-2	MutationTaster	CADD
*CDK9* (9q34.11)	NM_001261	c.862G>A, p.(A288T)	Exon 7	Maternal	A/A/A/A/A/A	0	0.0014	0.0018	6.98E-06	Damaging (0.001)	Probably damaging (1.000)	Disease causing	27.9
		c.907C>T, p.(R303C)	Exon 7	Paternal	R/R/R/R/R/R	0	0	0	0	Damaging (0.000)	Probably damaging (1.000)	Disease causing	28.4

^a^Databases include: in-house exome data (*n* = 218), Human Genetic Variation Database (HGVD; http://www.genome.med.kyoto-u.ac.jp/SnpDB/), Integrative Japanese Genome Variation Database (4.7KJPN; https://ijgvd.megabank.tohoku.ac.jp/), and Genome Aggregation Database (gnomAD; https://gnomad.broadinstitute.org/)

^b^We performed four in silico computational algorithms to evaluate the pathogenicity of missense variants as follows: SIFT (http://sift.jcvi.org/www/SIFT_seq_submit2.html); scores ≤ 0.05 and those >0.05 are assessed as damaging and tolerated, respectively. Polyphen-2 (http://genetics.bwh.harvard.edu/pph2/); scores were evaluated as 0.000 (benign) to 1.000 (probably damaging). MutationTaster (http://www.mutationtaster.org/); alterations are classified as disease causing or polymorphisms. CADD (http://cadd.gs.washington.edu/); scores > 20 indicates the 1% most deleterious possible substitutions in the human genome

^c^Human/cattle/mouse/rat/chicken/zebrafish *CDK9* orthologs. Accession numbers were NM_001261.4 (human), NM_001014935.2 (cattle), NM_130860.3 (mouse), NM_001007743.1 (rat), NM_001006201.2 (chicken), and NM_212591.1 (zebrafish)

## Discussion

We report a male patient with multiple anomalies involving the eyes, ears, cleft lip, and palate, and intellectual disability and biallelic variants in the *CDK9* gene in which one variant was derived from the mother and the other from the father. Both variants are extremely rare, highly conserved during evolution, present within the critical kinase domain. Actually, both variants’ kinase activities were reduced compared with WT CDK9. Before this case, a pair of cousins and three patients with the same homozygous nonsynonymous variant p.Arg225Cys in the *CDK9* gene have been reported to exhibit a similar combination of malformations reminiscent of the CHARGE syndrome [4, 5]. However, a causal relationship between *CDK9* and the CHARGE-like syndrome was not established. Documentation of new compound heterozygous pathogenic variants of the *CDK9* gene [p.(A288T) of maternal origin and p.(R303C) of paternal origin] in the current patient established *CDK9* biallelic mutations as a novel cause of human eye and ear diseases.

This new entity overlaps with the CHARGE syndrome, a classic syndrome with multiple malformations caused by *CHD7*. The shared features include ocular anomalies, microtia and hearing loss, cleft lip and palate, undescended testes, and intellectual disability. However, our patient did not have the major CHARGE-like symptoms of coloboma and choanal atresia. The current study showed that the ocular features included defects in the eyelids and nasolacrimal ducts, abduction of eye movement, cataracts, and progressive rod–cone retinal dystrophy that developed during early childhood (Supplementary Table 2). It is notable that the ocular phenotype of retinal dystrophy is never associated with the CHARGE syndrome [13].

It is also noteworthy that this newly identified condition was accompanied by various defects of the external ear and hearing loss and classified as branchial defects. Many of the syndromes characterized by multiple malformations, including Treacher Collins syndrome, are caused by defects in the polymerase II pathway, which is regulated by CDK9. The role of the integrity of the polymerase II pathway in the pathogenesis of this newly identified condition requires further investigation using model organisms.

The phenotypic similarity between the CHARGE syndrome and the *CDK9*-related disorder is currently unexplainable by molecular interaction between CHD7 and CDK9. Yet, differentiating the two entities is important for genetic counseling because of the difference in the inheritance modes. The CHARGE syndrome is an autosomal dominant condition and thus the recurrence risk for the parents will be low when the parents are unaffected, whereas the *CDK9*-related disorder is an autosomal recessive condition and thus carries a high risk of recurrence of 25% for the parents. The clinical distinction of the two may be possible through clinical evaluation of the joint contracture, which may be a characteristic feature of the *CDK9*-related disorder. The absence of finger flexion creases and large halluces as in the previously mentioned cousins and the current case are shared and are likely characteristic features of the *CDK9*-related disorder. Furthermore, ophthalmic evaluation is essential for these patients. The visual prognoses differ markedly between the two entities, in that retinal dystrophy is progressive in the *CDK9*-related disorder. One previous patient also was reported to have suspected retinal dystrophy [5]; however, none of the previous patients were examined by OCT and ERGs to determine the definitive pathogenesis.

In the current study, we investigated the detailed retinal structure and function using OCT, OCTA, and ERG and showed that an advanced stage of rod–cone dystrophy with abnormal foveal vascularity occurred in a child with biallelic *CDK9* mutations. Although the ocular phenotype varies, *CDK9* mutations may be involved not only in genetic programs for cellular death and differentiation during organogenesis but also in maintaining transcriptional homeostasis of the photoreceptor cells [14], which result in a vision-threatening phenotype of the early-onset severe retinal dystrophy.

In conclusion, *CDK9* biallelic variants cause a CHARGE syndrome-like malformation syndrome with contracture of the fingers and retinal dystrophy as distinguishing features.

## Materials and methods for Fig. 2e

### Autophosphorylation assays for CDK9 variants

Autophosphorylation activity of the CDK9 variants identified in reported individuals were evaluated in vitro. Human embryonic kidney 293 (HEK293) cells were transfected with the expression plasmids encoding Flag-tagged CDK9 protein or its mutants with cyclin T1 expression plasmids. Each fusion protein was expressed and immunoprecipitated with anti-Flag antibody. After purification, the protein was used as substrate for the following phosphorylation assay. Aliquots of immunoprecipitated CDK9 proteins were incubated for 90 min with or without 50 μM ATP in 40 μL of kinase buffer containing 40 mM Tris (pH7.5), 100 μM BSA, and 20 mM MgCl_2_ at 25 °C, and the ADP produced by autophosphorylation was measured using an ADP-Glo^®^ Kinase Assay kit (Promega). Immunoprecipitates from cells transfected with empty vector were used for measurement of background activity.

### Cell culture and transfection

HEK293 cells were grown in Dulbecco’s modified Eagle’s medium supplemented with 10% fetal bovine serum. HEK293 cells were transfected with the expression plasmids using Polyethylenimine MW 25000 (Polysciences, Warrington, PA).

## Supplementary information

Supp Table 1

Supp Table 2

Supp Figure 1
