# Supplementary material for: Biallelic CDK9 variants as a cause of a new multiple-malformation syndrome with retinal dystrophy mimicking the CHARGE syndrome
Source: J Hum Genet. 2021 Feb 27;66(10):1021–7. doi: 10.1038/s10038-021-00909-x (PMC8472910; doi:10.1038/s10038-021-00909-x)
Supplement: Supplementary file 3 — Supp Figure 1 [file 10038_2021_909_MOESM3_ESM.pptx]

## Slide 1
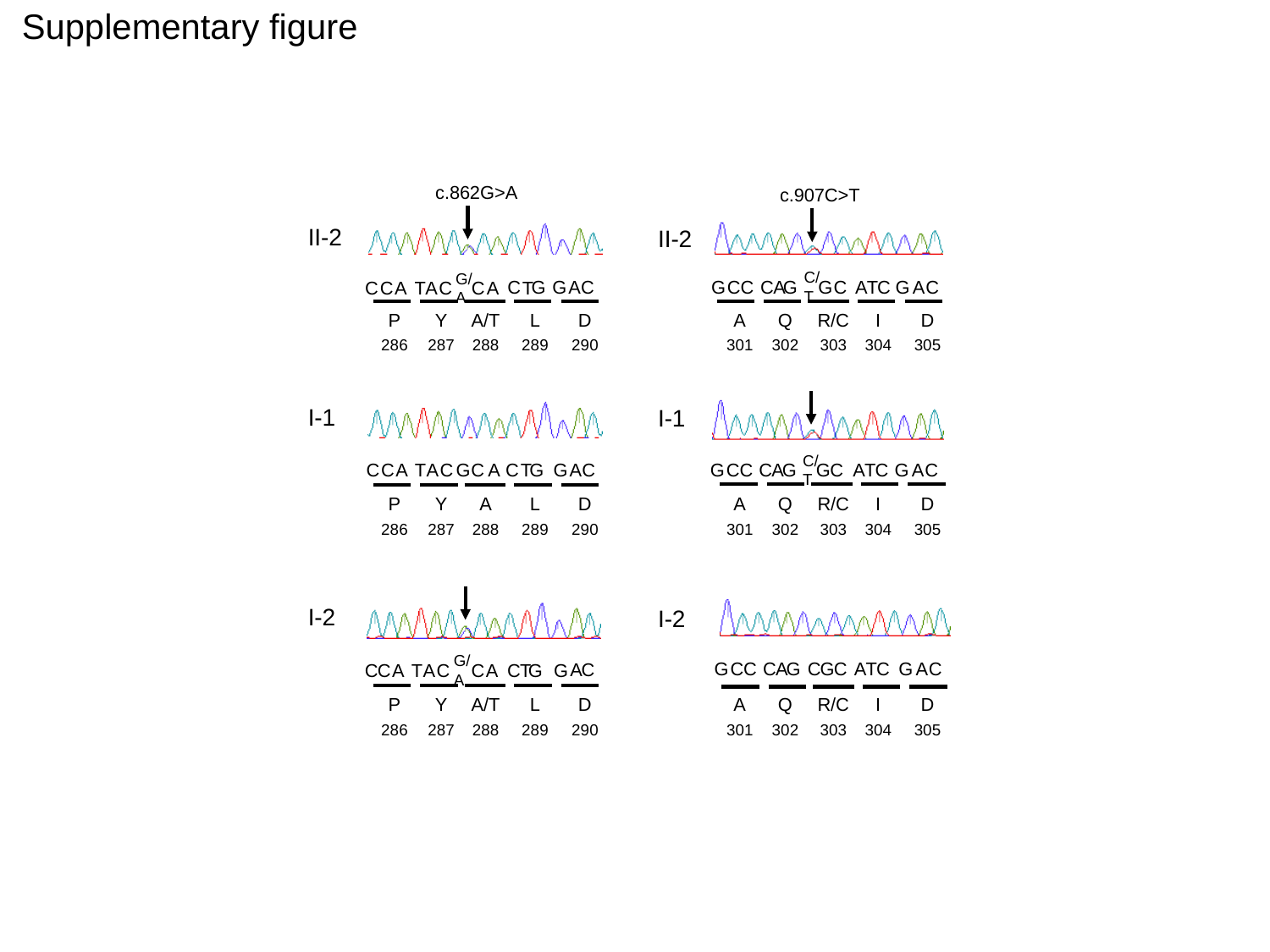

Supplementary figure
c.862G>A
c.907C>T
II-2
II-2
C/
T
G/
A
C
A
G
C
C
C
A
G
G
C
A
T
C
G
A
C
G
C
G
A
C
C
A
T
A
C
C
T
P
Y
A/T
L
D
A
Q
R/C
I
D
286
287
288
289
290
301
302
303
304
305
I-1
I-1
C/
T
G
C
C
C
A
G
G
C
A
T
C
G
A
C
C
C
A
T
A
C
G
C
A
C
T
G
G
A
C
P
Y
A
L
D
A
Q
R/C
I
D
286
287
288
289
290
301
302
303
304
305
I-2
I-2
G/
A
G
C
C
C
A
G
G
C
A
T
C
G
A
C
C
C
A
G
C
G
A
C
C
A
T
A
C
C
T
P
Y
A/T
L
D
A
Q
R/C
I
D
286
287
288
289
290
301
302
303
304
305
